# Natural compounds targeting major cell signaling pathways: a novel paradigm for osteosarcoma therapy

**DOI:** 10.1186/s13045-016-0373-z

**Published:** 2017-01-07

**Authors:** Pablo Angulo, Gaurav Kaushik, Dharmalingam Subramaniam, Prasad Dandawate, Kathleen Neville, Katherine Chastain, Shrikant Anant

**Affiliations:** 1 Division of Hematology and Oncology, Children’s Mercy Hospital, Kansas City, MO 64108 USA; 2 Department of Surgery, The University of Kansas Medical Center, 3901 Rainbow Boulevard, Mail Stop 3040, Kansas City, KS 66160 USA; 3 The University of Kansas Cancer Center, The University of Kansas Medical Center, Kansas City, KS 66160 USA; 4 Division of Hematology and Oncology, Arkansas Children’s Hospital, Little Rock, AR 72202 USA

**Keywords:** Osteosarcoma, Signaling pathways, Natural compounds, Ezrin

## Abstract

Osteosarcoma is the most common primary bone cancer affecting children and adolescents worldwide. Despite an incidence of three cases per million annually, it accounts for an inordinate amount of morbidity and mortality. While the use of chemotherapy (cisplatin, doxorubicin, and methotrexate) in the last century initially resulted in marginal improvement in survival over surgery alone, survival has not improved further in the past four decades. Patients with metastatic osteosarcoma have an especially poor prognosis, with only 30% overall survival. Hence, there is a substantial need for new therapies. The inability to control the metastatic progression of this localized cancer stems from a lack of complete knowledge of the biology of osteosarcoma. Consequently, there has been an aggressive undertaking of scientific investigation of various signaling pathways that could be instrumental in understanding the pathogenesis of osteosarcoma. Here, we review these cancer signaling pathways, including Notch, Wnt, Hedgehog, phosphatidylinositol-4,5-bisphosphate 3-kinase (PI3K)/AKT, and JAK/STAT, and their specific role in osteosarcoma. In addition, we highlight numerous natural compounds that have been documented to target these pathways effectively, including curcumin, diallyl trisulfide, resveratrol, apigenin, cyclopamine, and sulforaphane. We elucidate through references that these natural compounds can induce cancer signaling pathway manipulation and possibly facilitate new treatment modalities for osteosarcoma.

## Background

Osteosarcoma (OS) is the most commonly diagnosed primary bone malignancy, with an incidence of 0.2–3 cases/100,000 annually in children and 0.8–11 cases/100,000 in adolescents. The incidence peaks in the second decade of life. While only 20% patients present with metastasis that is clinically detectable, the majority of the remaining 80% are presumed to have undetectable micro-metastases at diagnosis [1]. The cancer can be found on the bone surface, within the bone, or in extraosseous sites, including the lung [2]. The etiology of OS remains uncertain despite advancements in molecular sciences. The only known environmental factor for OS is ionizing radiation [3]. OS is ranked among the leading causes of cancer mortality in the pediatric population [4]. Therapy includes preoperative chemotherapy, surgery, and postoperative chemotherapy (cisplatin, methotrexate, and doxorubicin). Additional chemotherapy (ifosfamide and etoposide) has been reserved for patients with high risk of metastatic disease. While chemotherapy has increased overall survival to 60–75%, survival rates have remained the same for the last 30–40 years [5]. Moreover, only 30% patients with metastatic OS achieve a 5-year event free survival [6].

The vast majority of OS appears to be sporadic, occurring in patients without common familial members affected. Nevertheless, there is growing support that the cancer is associated with the activation of numerous oncogenes, including cyclin D1, mouse double minute 2 homolog (MDM2), and c-Myc. Additional studies have demonstrated that various signaling pathways appear to be involved in the tumorigenesis of OS, including Wnt and PI3/Akt [7]. Hence, characterizing molecular targets that are specific for OS will be paramount for developing new strategies for treatment modalities.

**Fig. 1:**
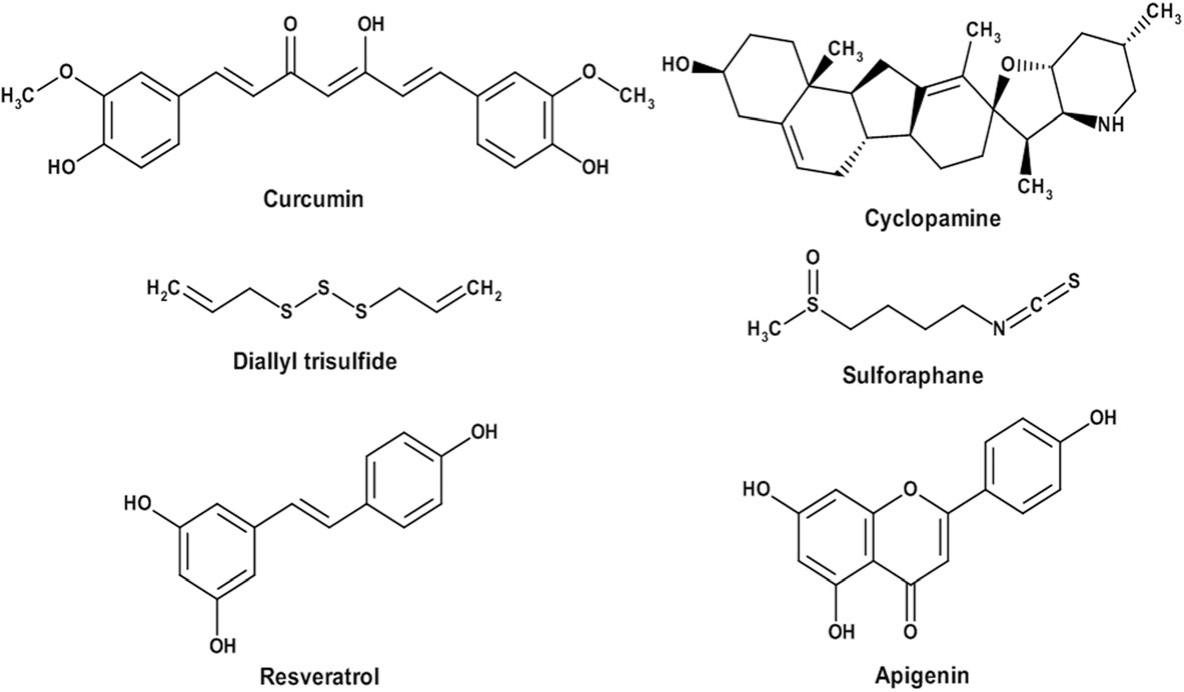
Chemical structure of the phytochemicals

Our group has been investigating key molecular signaling pathways that are integral in the origin, proliferation, and survival of osteosarcoma cells. Genes in these pathways are often mutated, resulting in activated cancer stem cells that proliferate without the normal regulatory mechanisms seen in noncancerous cells. In this review, we summarize a variety of signaling pathways that have demonstrated important roles in OS pathogenesis. In addition, we review numerous phytochemicals and inhibitors targeting these signaling pathways that show promising treatment abilities in OS. Table 1 lists these important cell signaling pathways along with the respective specific inhibitors. Figure 1 outlines the chemical structures of the six compounds described including curcumin, diallyl trisulfide, resveratrol, apigenin, cyclopamine, and sulforaphane. Through continuous research of these various pathways, an improved understanding of the molecular machinery promoting OS can be attained. Successful future treatment modalities depend on our ability to better understand and target these cellular pathways.

**Table 1:** Effect of Natural Compounds Targeting Major Stem Cell Signaling Pathways

Major signaling pathways	Compounds	Target	Effect	References
Notch	Curcumin	Downregulates transcription and translation Notch-1 and downstream genes Hes-1, Hey-1, and Hey-2 mRNA levels	^1^Induces apoptosis by increasing reactive oxygen species	[43, 55]
	Diallyl trisulfide	Targets Notch-1 intracellular domain	Decreases expression of Notch downstream genes. Increases expression of potential tumor suppressor micro RNAs (miR-143 and miR-145) and decreases tumor promoting micro RNA miR-21	
WNT/β-catenin	Resveratrol	β-catenin; histone H2AX	Apoptosis of OS cells by decreasing mRNA and protein expression of β-catenin and c-Myc Histone H2AX phosphorylation causes telomere instability and DNA damage	[24, 51, 54]
	Apigenin	β-catenin	Decreases protein expression of β-catenin and decreases matrix metalloproteinase 14 (MMP14) expression	
Hedgehog	Cyclopamine	Binds to SMO	Prevents signal transduction to GLIS	[30]
PI3/AKT	Sulforaphane	ERK and AKT	Suppresses ERK and AKT phosphorylation, induces apoptosisthrough G2/M phase arrest	[46]

## Signaling pathways

### Notch signaling

The Notch signaling pathway is an evolutionarily conserved pathway that plays an important role in embryonic and postnatal development in many organisms. The pathway is highly pleiotropic and affects vital processes of organ development as well as regulation of self-renewal of adult stem cells, thereby maintaining tissue homeostasis [8]. However, due to its multifunctional nature, this pathway is vulnerable to aberrant activation of signaling components and is associated with multiple human disorders, including various developmental syndromes and malignancies [8–10]. Therefore, in recent years, notch signaling has become one of the most important potential targets for developing novel therapeutic strategies.

Notch signaling is a multi-tiered, well-organized, tightly regulated cell/tissue-dependent cascade of signaling events. It requires various components for its maturation, activation, and execution. The Notch signaling family consists of four receptors, known as Notch-1 to Notch-4, and five DSL (Delta/Serrate/Lag-2) ligands, known as Jagged-1 and Jagged-2 (Jag-1 and Jag-2) and Delta-like-1, Delta-like-3, and Delta-like-4 (Dll-1, Dll-3, and Dll-4).

**Fig. 2:**
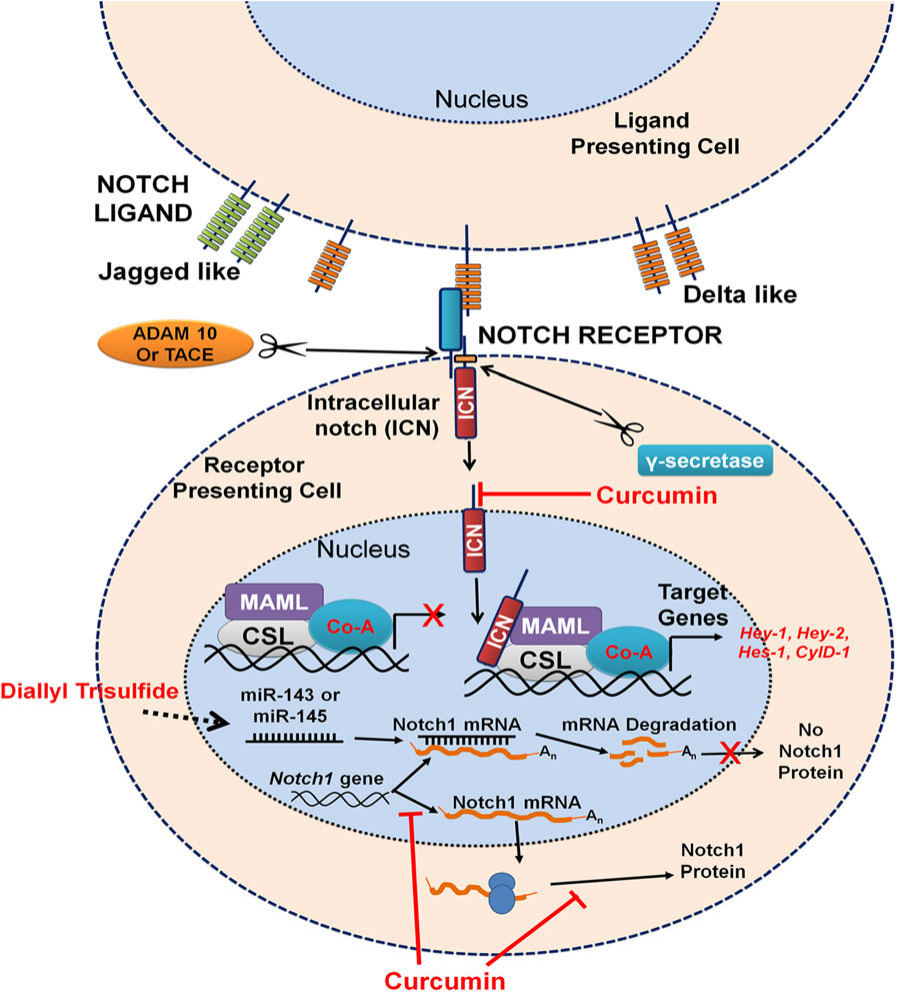
Notch signaling pathway. Ligand from the presenting cell binds to the notch receptor on the receiving cell. Notch extracellular truncated (NEXT) domain is cleaved by ADAM metalloprotease and γ-secretase yielding the notch intracellular domain (NICD). NICD is translocated to the nucleus where it complexes with transcription factor CSL 9 CBF1/suppressor of hairless/Lag 1 and transcriptional coactivator of the mastermind-like proteins (MAML). The complex can then activate target gene transcription. Diallyl trisulfide (DATS) treatment increases expression of tumor suppressor microRNAs: miR-143 and miR-145. MicroRNAs bind to Notch1 mRNA and results in mRNA degradation with no translation of Notch1 protein. Curcumin downregulates transcription and translation Notch1 and downstream genes Hes-1, Hey-1, and Hey-2 of the nucleus

Both the receptors and the ligands transmembrane proteins, and activation of the pathway occurs when a ligand from the neighboring cell interacts with the receptor [8, 11]. The interaction triggers conformational changes in the ligand-receptor complex that exposes an extracellular site on the notch receptor to proteolytic cleavage by tumor necrosis factor-alpha converting enzyme (TACE/ADAM17/CD156q), a component of the “a disintegrin and metalloprotease”, or ADAM (Fig. 2). A key regulatory step in notch activation and signaling, this cleavage generates the membrane-attached notch extracellular truncation (NEXT) fragment, which is located within the negative regulatory region (NRR) of the extracellular domain of the notch receptor. This NEXT fragment acts as a substrate for the γ-secretase protein complex, which consists of nicastrin, presenilin, presenilin enhancer-2 (PEN-2), and anterior pharynx defective 1 (APH1) [12, 13]. Once it is generated, NEXT is cleaved by γ-secretase to release and trigger translocation of the notch intracellular domain (NICD) into the nucleus. The active NICD can bind to mastermind-like proteins (MAML) and recombination signaling binding protein of hairless-J (RBPJ/CBF1) and form a nuclear activator complex to regulate transcription of downstream gene targets such as the hairy and enhancer of split (Hes) family of genes and the Hes-related family BHLH transcription factor with YRPW motif (Hey) family genes (Fig. 2). In the absence of NICD, RBPJ/CSL may associate with co-repressor proteins and repress transcription of target genes [14, 15].

Studies have documented the association of the notch signaling pathway with the resistance, aggressiveness, and metastatic potential of OS. This association has been validated in various experimental models including OS human/mouse cell lines, in vivo mice/canine models, and also in patient samples. All of these studies showed that OS cells with higher metastatic potential have higher basal levels of notch receptors, especially notch-1, notch-2, notch ligands (Dll-1/Jagged-1), and notch target genes such as hey-1 and hes-1, as compared to normal osteoblasts or non-metastatic OS cell lines. Higher expression levels of these notch signaling associated genes or proteins were shown to be involved in invasiveness and metastasis and thus in impacting OS patient survival [16, 17]. Increased levels of jagged-1 in OS cells promote bone metastasis by activating stromal notch signaling. IL-6 secretion from osteoblasts continues to augment tumor growth [18]. Notch signaling was also reported to be associated with increased aldehyde dehydrogenase (ALDH) activity, which results in an aggressive metastatic phenotype in murine OS cell lines (K7M2 and K12). K7M2 cells (highly metastatic in nature) showed upregulation of expression of notch signaling genes, including notch-1, notch-2, notch-4, and downstream targets genes, such as stat-3 and hes-1, compared to K12 cells. Elevated ALDH activity in K7M2 cells was abolished by inhibiting the notch signaling pathway and hence resulted in decreased metastatic behavior [19]. Therefore, abolishing expression of hey-1, hes-1, notch-1, and jagged-1 by using γ-secretase inhibitors (GSI) also abolished their direct/indirect effects on survival, bone metastasis, and invasiveness in OS [16, 18]. These findings suggest that inhibiting notch signaling at various points may be a novel therapeutic strategy for preventing OS invasiveness and metastasis [17].

In the last decade, several studies have demonstrated the role of microRNAs (miRNAs) in the progression, differentiation, and function of different cell types and in the pathogenesis of various human diseases. Recently, the expression pattern of miRNAs and their role in osteosarcoma was studied. It was observed that there was a significant increase in expression of some miRNAs (10-fold) in OS patients as compared to normal controls. Three of these miRNAs (miR-338-3p, miR-891a, and miR-199b-5p) were upregulated in OS cells. Further, ectopic expression of inhibitor of miR-199b-5p in OS cell lines showed a change in expression of notch pathway components and revealed that miR-199b-5p plays a role in notch signaling in OS [20]. Further work demonstrated that the expression of the microRNA 34 cluster (noted to downregulate Dll-1, notch 1, and notch 2) showed an inverse correlation with invasiveness in some osteosarcoma tumors, suggesting that this family of microRNAs may also be responsible for regulating notch expression in some tumors [21]. Additional studies have shown an association with Wnt signaling in the regulation of notch signaling in OS. In one study, Wnt10b expressing U2OS human OS (U2OS-Wnt10b) cells were compared to parental U2OS cells. In addition, differential expression of 1003 genes was compared. Genes involved in notch signaling (especially notch-1 and Jagged-1) were upregulated, whereas the notch inhibitor was significantly downregulated, leading to activation of the classic notch responsive genes (hes-1 and hey-1) [22]. These findings suggested that activation of Notch signaling plays a critical role in the pathogenesis of human OS and its inhibition could be a therapeutic approach for the treatment of this mesenchymal tumor [23].

### Wnt signaling

The Wnt signaling pathway is a highly conserved pathway responsible for a variety of functions including cell migration, cell fate determination, organogenesis, and stem cell renewal. Wnt signaling activates numerous transduction cascades in the cell. These cascades include Wnt/β-catenin dependent pathway and β-catenin-independent pathways. Alterations and dysregulation in the Wnt pathway can result in cancers of the skin, breast, and colon.

**Fig. 3:**
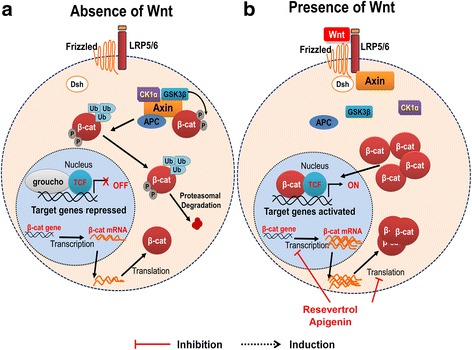
Wnt signaling pathway. **a** In the absence of the Wnt glycoprotein, β-catenin is degraded after being ubiquitinated and phosphorylated by the destruction complex. Target genes in the nucleus are not activated. **b** In the presence of Wnt, the glycoprotein binds to the extracellular transmembrane Frizzled receptor family (Fz and LRP5/6). Thereafter, the signal activates the protein Dishevelled (Dsh/DV1) in the cytoplasm. This binding results in disrupting the β-catenin destruction complex of various proteins including: axin, casein kinase 1α, adenomatous polyposis coli (APC), protein phosphatase 2A (PP2A), and glycogen synthase kinase 3 (GSK–3β). β-catenin translocates to nucleus where it can act as transcriptional coactivator of transcription factors of TCF/LEF family. Resveratrol and apigenin decrease protein expression of β-catenin

β-catenin is an integral protein in the Wnt signaling pathway that is responsible for regulating gene transcription and cell-to-cell adhesion. The level of the protein is stably maintained through degradation and phosphorylation. Mutations in β-catenin cause amino acid substitutions resulting in inappropriate phosphorylation of the protein. The phosphorylated protein is subsequently not recognized appropriately by the ubiquitin ligase E3. Hence, dysregulation of the Wnt pathway results in β-catenin accumulating without being degraded and then translocating to the nucleus where it activates transcription of oncogenes [24]. Expression of downstream genes includes *c*-*Myc*, *cyclin D1*, and *survivin* (an inhibitor of apoptosis). Wnt glycoproteins bind to the extracellular transmembrane Frizzled receptor family. Thereafter, the signal activates the protein Dishevelled (Dsh/DV1) in the cytoplasm. Wnt can then branch into three different signal cascades: canonical, non-canonical planar cell polarity, and non-canonical Wnt/Ca^2+^ [25]. The hallmark of the canonical pathway is the translocation of β-catenin from the cytoplasm to the nucleus where it acts as a coactivator of transcription factors of TCF/LEF family (Fig. 3b). Without the Wnt glycoprotein binding to the Frizzled receptor, β-catenin would be degraded by a β-catenin destruction complex. This degradation complex results in the phosphorylation of β-catenin at various sites mediated by the scaffolding protein Axin which can interact with glycogen synthase 3β (GSK3), Casein kinase 1 alpha 1 (CK1α), and β-catenin. The phosphorylation of β-catenin comes by way of CK1α at serine 45 and by GSK3 at threonine 41, serine 37, and serine 33. Those final phosphorylation sites at serine 33 and 37 form a binding site for beta-transducin repeat-containing E3 ubiquitin protein ligase (β-Trcp) which can then degrade β-catenin [26] (Fig. 3a). The hallmark of planar cell polarity is actin cytoskeleton regulation. This pathway is responsible for organizing sensory cilia of the inner ear as well as organizing hair follicles. The crux of the Wnt/Ca^2+^ pathway is the stimulation of intracellular calcium release from the endoplasmic reticulum by way of interaction with G proteins. This pathway is important for dorsal axis formation and regulation of tissue separation. β-catenin is not involved in either non-canonical pathway [25].

Wnt is known to play an important role in osteoblastogenesis. Because osteosarcoma cancer cells are believed to be derived from osteoblasts, it is reasonable to postulate that antagonizing the Wnt pathway might yield inhibition of osteosarcoma cells as osteoblastogenesis is impaired. Wang et al. reported that the chemotherapeutic docetaxel could successfully inhibit the proliferation of two osteosarcoma cancer cell lines, U2OS and SaOS-2, in a time-dependent and dose-dependent manner by interfering with the Wnt pathway. Docetaxel functioned by inhibiting the transcriptional activity of β-catenin [27]. Zhao et al. also demonstrated that the Wnt pathway could be targeted by utilizing naked cuticle homolog-2 gene (*NKD2*), which encodes a protein that serves as a negative regulator in the Wnt pathway. In a mouse model, NKD2 was overexpressed with osteosarcomas, and the ability of the cancer cells to proliferate, invade, and metastasize was markedly decreased. Evaluation of the tumors with NKD2 overexpression revealed downregulation in molecules required for angiogenesis and upregulation of tumor suppressor genes [28].

Wnt signaling pathway has also demonstrated to be involved with the oncogene v-maf avian musculoaponeurotic fibrosarcoma oncogene homolog K (MAFK), a homolog that is integral in cell proliferation in vitro. Using a gene microarray, Wang et al. showed that the oncogene expression level of MAFK could be induced by Wnt-1. Hence, the Wnt-1 induction of the expression of MAFK resulted in a significant increase in the cell viability, further demonstrating the role of Wnt in osteosarcoma pathogenesis [29]. These experiments provide evidence that antagonizing the Wnt pathway could have some therapeutic efficacy in osteosarcoma treatment.

### Hedgehog signaling

**Fig. 4:**
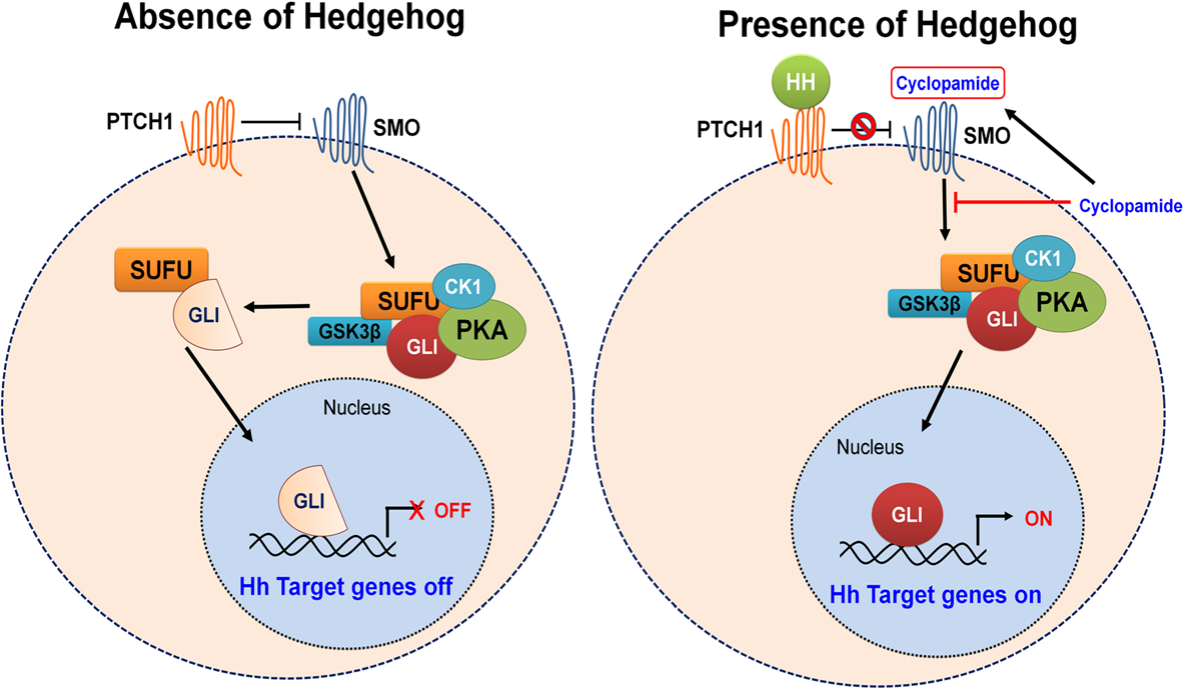
Hedgehog signaling pathway. **a** In the absence of Hh ligand, PTCH prevents activation of SMO. SMO cannot inhibit protein kinases including PKA, GSK-3β, and CK1. These protein kinases phosphorylate GLI protein in complex with SUFU resulting in cleavage of GLI into a repressed form. The repressed form will translocate to the nucleus inhibiting Hh target gene expression. **b** Hh ligand binds PTCH1 (transmembrane receptor). Smoothened (SMO) is relieved and inhibits proteolytic cleavage of GLI protein resulting in an active form. The active GLI protein translocates to nucleus and activates transcription factors. Cyclopamine binds to SMO preventing signal transduction to GLIS

It has been estimated that 70% of OS specimens possess hedgehog (Hh) signaling components. Hedgehog was discovered as a critical factor in the development and progression of multiple cancers. The pathway is unique in that it is comprised of both tumor suppressor genes and oncogenes. The signaling pathway is associated with three ligands: Sonic hedgehog (SHh), Indian hedgehog (IHh), Desert hedgehog (DHh), and additional components of the pathway include 12-transmembrane Patched proteins (PTCH1 and PTCH2), 5-zinc finger transcription factors GLI1, GLI2, GLI3 (glioma-associated oncogene homologs), and the 7-transmembrane protein smoothened (SMO). In the canonical pathway (beta catenin dependent), a ligand will bind to PTCH1 (a transmembrane receptor), which relieves SMO (G-protein coupled receptor-like protein). SMO in turn can activate downstream transcription factors called GLI family zinc finger proteins (Fig. 4b). If ligands are not present, PTCH will block the entry of SMO. As a result, SMO is not able to functionally inhibit protein kinases including PKA, GSK-3b, and CK1. Hence, the protein kinases can phosphorylate GLI proteins in complex with SUFU and cause proteolytic cleavage of GLI. The cleavage of GLI results in the formation of a repressed form of GLI that will translocate to the nucleus and turn off signaling (Fig. 4a). The usual Hh target genes include transcription factors such as cyclin D1, B cell CLL/lymphoma 2 (BCL2), and vascular endothelial growth factor (VEGF). Lo et al. evaluated Hh pathway in 42 human OS samples and found higher expression levels of genes encoding IHH, PTCH1, and GLI genes in the tumors. It is speculated that high levels of IHh result in a larger tumor size [30].

Other research has demonstrated that SMO and GLI activation are vital for OS progression. Hirotsu et al. determined that SHh, DHh, PTCH1, GLI1, GLI2, and SMO were overexpressed in five different OS cell lines (NHOst, 143B, HOS, MG63, and NOS-1). It was speculated that the promoter of GLI1 was inactivated in human OS specimens and that GLI2 mediated the activity of downstream SMO; thus, GLI1 is downregulated in OS while GLI2 is upregulated [30]. Nagano et al. showed that GLI2 was involved in the tumor invasion and metastasis. In mice that had GLI2 knocked down via transfection of GLI2-shRNA, tumor growth was decreased compared to mice that did not have GLI2 knocked down [30].

Drug discovery for the Hh pathway has been concentrated on targeting SMO, which serves as the primary transducer in Hh signaling. When SMO is inhibited, the transcription factors GLI1 and GLI2 remain inactive. This inactivity prevents the expression of tumor-activating genes. As a result of inhibiting SMO, apoptosis and growth arrest of OS cells occur in vivo and in vitro [31]. Hh inhibitors including the plant-derived cyclopamine and its respective derivatives such as saridegib and vismodegib are potentially promising drug options. However, more research is needed to explore the broad biological effects of inhibiting the Hh pathway [30].

### PI3K-AKT-mTOR and Ras-Raf-MEK-ERK pathways

**Fig. 5:**
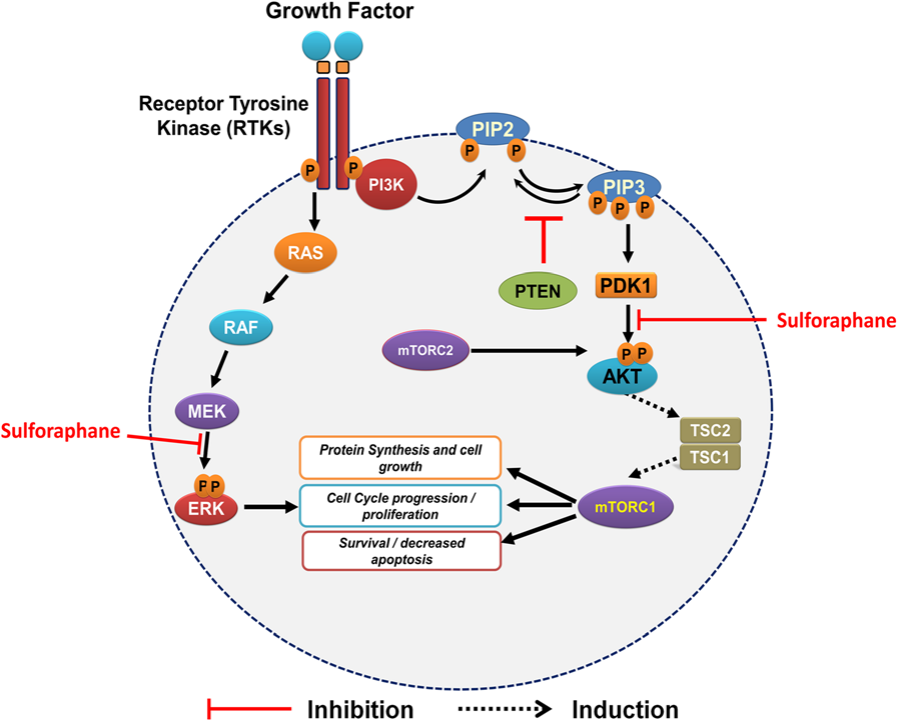
PI3K-AKT-mTOR and RAS-RAF-MEK-ERK pathways. Growth factor binds to epidermal growth factor receptor and can proceed by two different pathways. For the PI3 pathway, the ligand activates tyrosine kinase receptor activity resulting in phosphorylation of receptor. PI3K binds to phosphorylated receptor and becomes activated. PI3K then binds to PIP2 on inner membrane and phosphorylates PIP2 to PIP3. PIP3 activates AKT via PDK1. AKT can then phosphorylate and activate protein mTOR which results in cell growth, cell proliferation, and cell survival. For the RAS-RAF pathway, growth factor binding to tyrosine kinase receptor activates RAS which in turn activates RAF. RAF activates MEK which phosphorylates ERK to decrease apoptosis and increase cell proliferation and growth. The compound sulforaphane suppresses the phosphorylation of AKT and ERK

PI3Ks are a lipid kinase family that can be categorized into three different classes based on homology and the particular substrate they bind. Class I lipid kinase is most often associated with cancer and is a heterodimer consisting of a regulatory subunit and a catalytic subunit. When the catalytic subunit is activated, phosphatidylinositol 4,5-biphosphate (PIP2) is altered to phosphatidylinositol 3,4,5-triphosphate (PIP3). PIP3 is then able to recruit signaling proteins including phosphoinositide-dependent kinase 1 (PDK1) and AKT. AKT is partially activated by PDK1 at threonine 308 (Thr308). Thereafter, AKT is fully activated at serine 473 (Ser473) by the following proteins: integrin-linked kinase (ILK), DNA-dependent protein kinase (DNA-PK), mTORC2, and even AKT itself. Now fully activated, AKT can translocate to the nucleus from the membrane and the cytoplasm, where it can phosphorylate or activate downstream targets [32] (Fig. 5).

A key downstream target of the PI3/AKT pathway is mammalian target of rapamycin (mTOR). The mTOR is a serine/threonine kinase that regulates protein synthesis and cell cycle progression. mTOR has two forms known respectively as mTORC1 and mTORC2. mTORC1 controls autonomous growth, while mTORC2 mediates cell survival and proliferation. mTORC1 is integral in the carcinogenesis of a multitude of cancers including OS. mTORC1 is made of proline-rich AKT substrate (PRAS40), DEP domain-containing mTOR-interacting protein (Deptor), a regulatory-associated protein of mTOR (Raptor), and mammalian LST8/G-protein β-subunit like protein (GβL). When activated, mTORC1 can mediate the phosphorylation of ribosomal protein S6 kinases (S6K) as well as the eukaryotic translation initiation factor 4E-binding protein 1 (4E-BP1). Phosphorylation of 4E-BP1 causes the release of eukaryotic translation initiation factor 4E (eIF4E), which leads to the translation of protein as well as cell cycle progression [7]. mTORC2 consists of mTOR, LST8/G-protein β-subunit-like protein (GβL), Rictor, and mammalian stress-activated protein kinase-interacting protein (mSIN1) and is an activator of AKT enabling cell survival [7]. mTORC2 was found to also directly phosphorylate PI3K. This phosphorylation is necessary to maximize the activation of the anti-apoptosis kinase and enhance cell proliferation, migration, and survival. This discovery has led to the development of small molecular mTOR inhibitors [33]. The tumor suppressor tuberous sclerosis complex (Tsc) 2 can be phosphorylated by AKT. This phosphorylation inhibits the formation of Tsc1/Tsc1 heterodimers. Inhibition of Tsc1/Tsc2 heterodimers preserves an active GTP-bound state of the protein Rheb (Ras homolog enriched in brain) and leads to an increase in mTORC1 activity [7].

Other additional important targets that are affected by AKT include the following: GSK3β, nuclear factor-κB (NF-κB), forkhead box O1 (FOXO1), and apoptotic factors such as Bax and B cell lymphoma 2 (BCl-2). Cyclin D1 and the oncoprotein c-Myc are upregulated after the deactivation of GSK3β. The inhibition of FOXO1 by AKT accelerates the cell cycle by downregulating cyclin-dependent kinase inhibitors p27 and p21. The central signaling factor NF-κB is activated by AKT and is a main signaling factor that allows cancers to develop and progress and acquire drug resistance in aggressive malignancies. AKT decreases the proapoptotic levels of Bad and Bax and conversely increases the anti-apoptotic levels of Bcl-2, Bcl-xl, and myeloid cell leukemia 1 (Mcl1). Furthermore, Akt reduces the release of tumor suppressor p53. The generalized function of PI3K/AKT signaling pathway is to minimize apoptosis while increasing cellular proliferation and survival [7].

Targeting the AKT pathway was demonstrated when Lu et al. utilized the phytoestrogen 5,7-dihydroxy-4′-methoxyisoflavone to induce apoptosis in OS cells. The phytoestrogen did not have any effect on the normal human skin fibroblasts but did selectively inhibit the U2OS cancer cells. The inactivation of the pathway was confirmed by downregulation of BCL-2 and the upregulation of the expression of Bax [34].

H2 relaxin (RLN2) is a peptide hormone and member of the insulin-like superfamily that has been shown to play a role in the pathogenesis of OS by positively regulating the AKT pathway. Ma et al. demonstrated that overexpressing RLN2 increased OS cellular invasion and migration, while silencing RLN2 decreased the ability of these cells to invade and survive. Moreover, the OS cells were more sensitive to cisplatin chemotherapy when RLN2 was silenced. Western blot analysis supported the positive direct correlation of RLN2 and AKT, showing decreased signal intensity of AKT when RLN2 was inhibited. These results illustrate the importance of modulating the signaling pathway AKT in the treatment of OS [35].

The Ras-Raf-MEK-ERK (along with PI3/AKT) is the most altered signaling pathway in solid tumor cancers. The pathway commences with the binding of growth factors to their receptors which activate the Shc/Grb2/SOS coupling complex. The complex subsequently activates the inactive protein Ras which modifies guanosine diphosphate (GDP) to guanosine triphosphate (GTP). A downstream target for RAS is RAF kinase. RAF kinase activates MEK1/2 which will catalyze the activation of ERK1/2. ERK1/2 in turn can phosphorylate numerous downstream targets integral in cell differentiation, proliferation, angiogenesis, and survival [36] (Fig. 5).

The role of MEK in osteosarcoma invasiveness was supported when Ye et al. demonstrated that overexpression of MEK was associated with osteosarcoma growth and metastasis. IHC showed positive expression of total protein for AKT, p38 MAPK, IGF-1R, and MEK in orthotopic mouse primary tumor. However, only phosphorylated MEK was seen via immunohistochemistry in both the primary as well as the metastatic tumor. Furthermore, when the MEK pathway was targeted with the MAPK/ERK inhibitor U0126, there was a decreased invasive ability of the OS cells in vitro. Hence, new targeted therapies could be implicated for the Ras/Raf/MEK/ERK signaling pathway which potentially could impair the invasiveness of OS [37].

### Ezrin

**Fig. 6:**
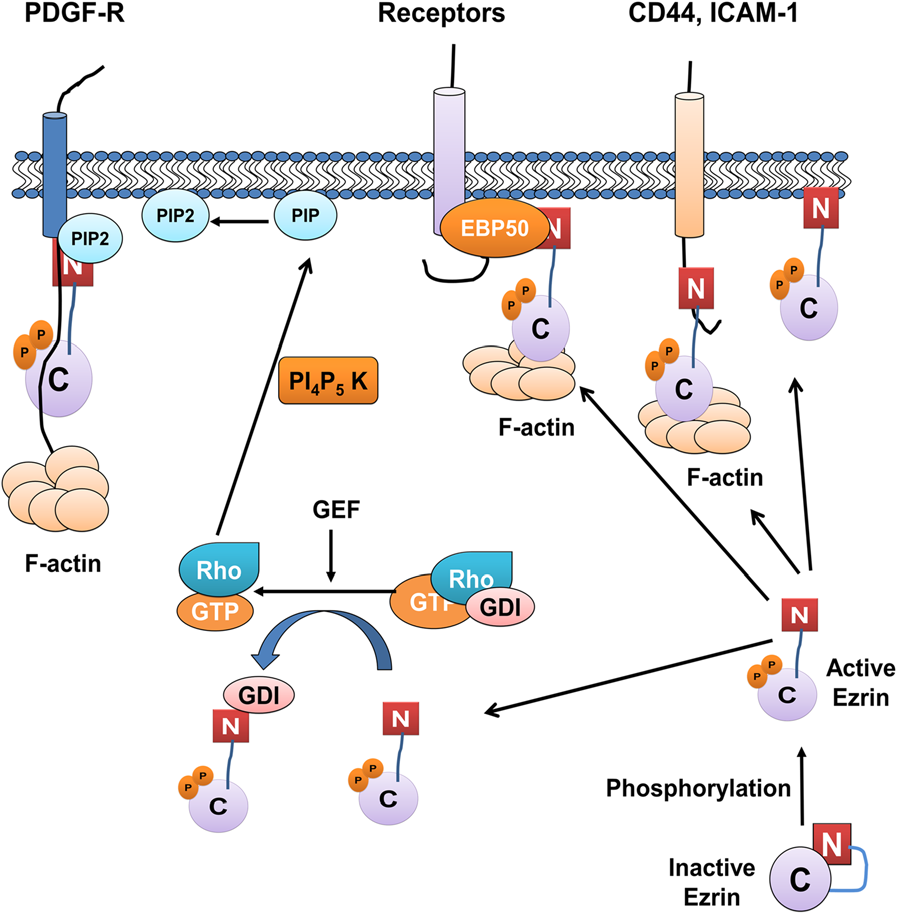
Ezrin pathway. While in the dormant form, ezrin with N-terminal ezrin/radixin/moesin (ERM)-associated domain (N-ERMAD) associates in the cytoplasm with carboxy-ERMAD (C-ERMAD). Ezrin then becomes phosphorylated at various sites resulting in transformation to the active form. The C-terminal of transmembrane proteins as well as C-ERMAD binds with the N-terminal of activated ezrin. In addition, ezrin can serve as a linker protein between specific membranous proteins and F-actin via ERM-binding phosphoprotein 50 (EBP50). Guanosine diphosphate inhibitor (GDI) from the Rho-GDI complex is displaced by activated ezrin. This displacement can then stimulate PI4P5 kinase activity which is catalyzed by GDP/GTP exchange factor (GEF). Thereafter, PI4P5 kinase can act on PIP to convert PIP to phosphatidylinositol (4,5)-bisphosphate (PIP2). Thus, PIP2 sequentially converts dormant ezrin into the active form

Gaining an improved understanding of metastasis involves identifying associated molecules and pathways that regulate cell motility and invasion. A family of proteins known as ERM (ezrin, radixin, and moesin) plays an important role in linking the actin cytoskeleton and the plasma membrane of a cell. Ezrin maintains cell motility, promotes cell invasion, and maintains cell adhesion. While in the dormant form, ezrin with N-terminal ezrin/radixin/moesin (ERM)-associated domain (N-ERMAD) associates in the cytoplasm with carboxy-ERMAD (C-ERMAD). Ezrin then becomes phosphorylated at various sites resulting in transformation to the active form. The C-terminal of transmembrane proteins as well as C-ERMAD binds with the N-terminal of activated ezrin. In addition, ezrin can serve as a linker protein between specific membranous proteins and F-actin via ERM-binding phosphoprotein 50 (EBP50). Guanosine diphosphate inhibitor (GDI) from the Rho-GDI complex is displaced by activated ezrin. This displacement can then stimulate PI4P5 kinase activity which is catalyzed by GDP/GTP exchange factor (GEF). Thereafter, PI4P5 kinase can act on PIP to convert PIP to phosphatidylinositol (4,5)-bisphosphate (PIP2). Thus, PIP2 sequentially converts dormant ezrin into the active form [38] (Fig. 6). Ezrin was discovered to be integral in OS and metastasis due to its ability to drive tumor progression by allowing OS metastatic cells to overcome a variety of stresses. A significant stress factor is the ability of OS cells to adapt to the new microenvironment of the secondary metastatic location in order to survive. The cells must first detach from the primary tumor, intravasate into the bloodstream, transport to the metastatic secondary site, and colonize and repopulate there.

A correlation appears to exist between high levels of ezrin expression in highly invasive cancer cells and low levels of ezrin expression in low-invasive cancer cells. In vivo experiments revealed that when ezrin was knocked down, cell invasion and migration were reduced. In contrast, when ezrin was overexpressed, cancer cells had higher ability to invade and migrate. Interestingly, ezrin expression was decreased when microRNA-183 (miR-183) was increased. Ezrin also appeared to show a correlation with N-cadherin expression. N-cadherin expression is associated with epithelial-to-mesenchymal transition (EMT). This particular cadherin is necessary for metastasis and cancer growth. This mechanism promotes detachment of the cancer cells from the primary site and facilitates the migration to blood vessels and secondary sites. Experiments have validated that ezrin results in increased N-cadherin expression [39].

Zhang demonstrated that ezrin ectopic overexpression in the MG63 OS cell line resulted in increased tumor migration and cell invasion in vitro. Ezrin’s effect on OS was further demonstrated in vivo through an experimental metastasis model in which the MG63 OS cells were delivered to female mice to develop pulmonary metastasis. Ezrin was notably found to increase N-cadherin and enhance the expression of the MAPK/ERK signaling pathway. Ectopic overexpression of ezrin in the OS cell line MG63 promoted tumor cell invasion and migration. Consistent with this finding, knockdown of ezrin inhibited tumor cell invasion and migration. Collectively, these results suggest that increased N-cadherin and ERK signaling activation by ezrin can promote aggressiveness in OS [40].

A meta-analysis was conducted to evaluate the expression level of ezrin in osteosarcoma patients compared to patient prognosis. Evaluation of 459 patients revealed higher frequency of ezrin expression in stage III and stage IV than in lower histological stages of osteosarcoma. Positive expression of ezrin correlated with lower overall survival [41]. Compounds that are able to successfully inhibit ezrin are being researched to serve as potential therapeutics for osteosarcoma.

One compound known as NCS305787 was discovered to directly bind to ezrin [42], inhibiting its function of invasive promotion. NSC305787 has a structure very similar to quinolone-containing compounds such as anti-malarial agents. On the basis that ezrin likely has a key role in the pathogenesis of malaria, additional anti-malarial compounds were screened to identify novel ezrin inhibitors with better efficacy and drug properties than NSC305787. One such compound, MMV667492, had improved physicochemical properties for drug likeness compared to NSC305787 and exhibited potent anti-ezrin activity in biological assays. The drug-like compounds MMV020549 and MMV666069 also showed promising activities in functional assays. Both compounds demonstrated superior activity compared to the NSC305787, especially in inhibiting pulmonary metastatic growth. These data demonstrate that anti-malarial compounds warrant further study in randomized clinical trials of OS [42].

### JAK/STAT pathway

Osteosarcoma has a predilection for the metaphyseal regions of the long bones, regions known to represent a large pool of mesenchymal cells. Several studies have reported that the stem cells can induce pro-inflammatory effects through the activation of multiple factors [43]. This inflammation yields bioactive molecules including growth factors and cytokines that are able to stimulate persistent cellular proliferation and subsequent malignant transformation. Interleukin-6 (IL-6), believed to be one of the most important inflammatory factors involved in this inflammatory process, can activate Janus tyrosine kinase (JAK) family members. These kinase family members including JAK1, JAK2, and tyrosine kinase 2 (TYK2) can in turn activate transcription factors of the signal transducer and activator of the transcription (STAT) family [44].

**Fig. 7:**
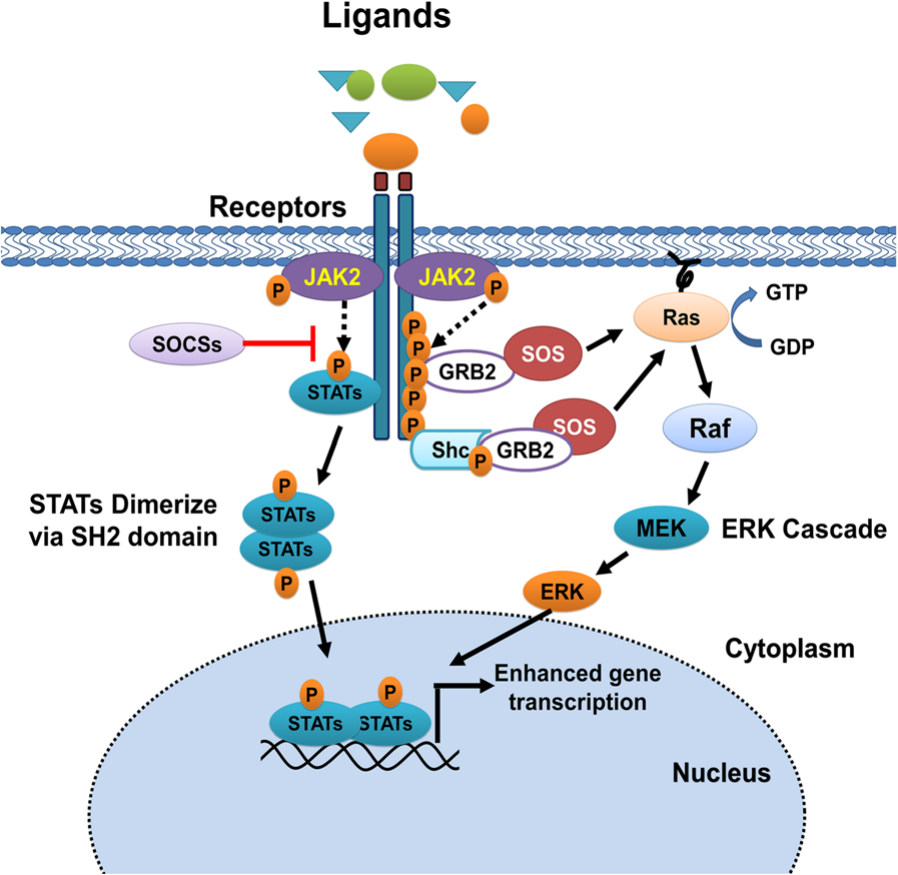
Jak/STAT pathway. The corresponding cytokine or growth factor ligand binds to the cell surface receptor prompting activated JAK2 protein to phosphorylate tyrosine residues in the cytoplasmic domain of the receptor. More so, JAK2 also phosphorylates recruited signal transducer and activator of transcription (STAT) which results in STAT dimerization via conserved Src homology 2 (SH2) domains. STAT dimers then translocate to the nucleus where they induce transcription of target genes. In addition, JAK acts as a docking site for SH2 containing adapter proteins including Src homology 2 domain-containing phosphatase 2 (SHP2), growth factor receptor bound protein-2 (GRB2), and Src homology 2 domain-containing transforming protein (SHC). GRB2 which is associated with Son of Sevenless (SOS) can bind the tyrosine phosphorylated receptor directly or indirectly by way of the Src homology 2 domain-containing protein (SHC). This binding results in the translocation of SOS to the membrane. At the membrane, SOS exchanges GDP for GTP on Ras guanine nucleotide-binding proteins. Ras-GTP can then activate MAPK cascade. Aside from RAS, JAK/STAT also interacts with PI3 and AKT pathways [65]. Under normal conditions gene expression is regulated by negative feedback mechanisms including the production of the negative regulator suppressors of cytokine signaling (SOCS)

The corresponding ligand binds to the cell surface receptor prompting activated JAK2 protein to phosphorylate tyrosine residues in the cytoplasmic domain of the receptor. More so, JAK2 also phosphorylates recruited STAT which results in STAT dimerization via conserved Src homology 2 (SH2) domains. STAT dimers then translocate to the nucleus via nucleoprotein interactor 1 (NP-1) where they induce transcription of target genes. In addition, the JAK/STAT also interacts with RAS/MAPK, PI3, and AKT pathways (Fig. 7). Under normal conditions, gene expression is regulated by negative feedback mechanisms including the production of the negative regulator suppressors of cytokine signaling (SOCS) [45].

IL-6 wields its pro-proliferative effect by binding to the IL-6 receptor complex. This complex is comprised of either IL-6R and glycoprotein 130 (gp130) or the soluble form of IL-6R (sIL-6R). When IL-6 binds to its respective receptor, the gp130 undergoes a conformation change and gp130 can then activate signal transducer and activator of transcription (STAT3). Mounting evidence is showing that constitutively activated STAT3 in the face of abnormal dysregulation promotes the development of tumors.

Tu et al. demonstrated that a neutralizing antibody could block the activation of STAT3 in OS cells [43] by way of a compound known as AG490 which is a specific and potent inhibitor of JAK2. By inhibiting JAK2, STAT3 could not be activated via phosphorylation, and thus, there was a reduction in the proliferation, migration, and invasion of OS cells. The effects were also seen in vivo in a nude mouse model injected with OS cell line Saos-2 and then treated with AG490 (Jak2 inhibitor). There was a significant reduction in tumor growth in those mice treated with AG490 [43]. Tu et al. further demonstrated a reduction in pulmonary metastasis and an overall increased survival in the mice. Thus, AG490 could be a potent inhibitor of OS cells [43].

## Chemopreventive agents and anti-cancer compounds

### Curcumin

Curcumin is a naturally occurring compound derived from the rhizomes of *Curcuma longa*. A member of the ginger family, it is a spice that has been commonly used for food preservation as well as for health care, primarily on the Indian subcontinent. The compound was first isolated two centuries ago, and has been used to treat a variety of systemic diseases, including pulmonary, dermatological, and gastrointestinal system disorders. Curcumin has been able to perform many of these functions because it possesses a wide array of functional characteristics including antioxidant, antiviral, antifungal, antibacterial, anti-inflammatory, and anti-cancer properties [46].

The anti-cancer properties of curcumin have been demonstrated in multiple types of cancers, including OS. Chang et al. evaluated the cytotoxicity of various concentrations of curcumin in the OS cell line MG63. The results demonstrated that the osteoblasts maintained 80% viability with all the curcumin concentrations, implying less sensitivity of the osteoblasts to the curcumin. MG63 had 50% cell viability with 10 μM curcumin compared to the control osteoblasts, suggesting increased sensitivity of OS to curcumin. Our own experimentation has demonstrated that curcumin can effectively target stem cells in patient-derived OS tumor samples resulting in reduced osteosphere formation status post treatment. In addition, live fluorescent staining of patient-derived OS cells treated with increasing concentration of curcumin demonstrated propidium iodide staining the cells’ nuclei representing cell death. These studies therefore suggest that curcumin can selectively kill malignant OS cells rather than healthy osteoblasts [47].

Additional studies have characterized how curcumin affects OS cancer cells. Li et al. showed that curcumin inhibited proliferation, activated apoptosis, induced G2/M phase cell cycle arrest, and decreased the ability of OS cells to invade and metastasize. These actions were accomplished by downregulating Notch-1 and the respective downstream genes, including Hes-1, cyclin D1, matrix metallopeptidase 2 (MMP-2), and matrix metallopeptidase 9 (MMP-9) (Fig. 2). This research provided the first evidence that Notch-1 and its respective downstream genes are downregulated in response to curcumin and presented the possibility that curcumin may be an effective compound for treating OS [48].

Chang et al. observed that curcumin was able to induce apoptosis in osteosarcomas by increasing the reactive oxygen species (ROS) in a dose-concentration-dependent manner. Furthermore, high concentration doses of curcumin (80 μM) led to the release of cytochrome C and activation of caspase-3, prompting apoptosis of MG63 cells [49].

Unpublished data from our lab confirm curcumin’s dose-dependent inhibitory effects on the proliferation of OS cells MG63, KHOS, and SJSA. Furthermore, curcumin in combination with traditional agents including doxorubicin and cisplatin results in a higher reduction of OS cells compared to the traditional chemotherapy alone. Therefore, curcumin appears to have potent anti-cancer activity and could be a novel agent to introduce in upfront therapy.

### Diallyl trisulfide

Numerous approaches are being used to study the effects of Notch signaling inhibition on many cancer types. These approaches include use of a neutralizing antibody against target proteins, use of dominant-negative mutant for key proteins in Notch signaling, and use of natural synthetic compounds to target Notch signaling. Inhibition of cancer progression by natural or synthetic compounds offers significant promise for reducing cancer incidence and mortality in patients. Use of diallyl trisulfide (DATS), an organosulfide derived from garlic, showed inhibition of proliferation in OS cells by triggering cell cycle arrest and apoptosis in vitro. DATS also has been reported to suppress cell survival, wound-healing capacity, invasion, and angiogenesis in OS cells through decreased expression of Notch-1 downstream genes, such as vascular endothelial growth factor (VEGF) and matrix metalloproteinases. DATS had contrasting effects on various microRNA. Treatment with DATS decreased tumor promoting miR-21 and increased potential tumor suppressor miR-143 and miR-145 (Fig. 2). These results suggest that DATS inhibited osteosarcoma growth and aggressiveness via a mechanism targeting a Notch-miRNA regulatory circuit [50].

### Resveratrol

Resveratrol (3,5,4′-trihydroxy-trans-stilbene) is a natural phenol and phytoalexin produced by several plants in response to injury or pathogens. Food sources include mulberries, raspberries, blueberries, grapes, and peanuts [51]. It was first isolated from roots of the white hellebore (*Veratrum grandiflorum* O. Loes). The phenol is an active constituent of the roots from *Polygonum cuspidatum* [52]. Historically, resveratrol has been reported to cause cell cycle arrest, promote apoptosis, and inhibit cancer cell proliferation in oral squamous carcinoma, glioblastoma, liver carcinoma, non-melanoma skin cancers, and thyroid carcinoma [53]. Rusin et al. reported that resveratrol inhibited cell growth and induced senescence in OS cells (U2-OS) by modifying the DNA metabolism. Resveratrol can alter the localization and expression of critical proteins integral in cell cycle regulation and DNA repair, as well as generate instability of the telomeres and promote DNA damage. Data demonstrated that OS cell growth was inhibited at 50-μM concentration, and the cells were arrested in the S phase of the cell cycle (suggesting interference with the metabolism of DNA) [54].

Zou et al. reported that resveratrol inhibited the proliferation of MG63 OS cells by downregulating β-catenin in the canonical WNT signaling pathway. Western blot and RT-qPCR determined that the protein and mRNA expression levels of β-catenin and C-myc were significantly downregulated (Fig. 3b). Additional experiments with animal models will need to be performed to confirm this effect. Nevertheless, this study indicates a potential treatment for OS through the natural compound resveratrol [24].

### Apigenin

Apigenin (4′,5,7-trihydroxyflavone) is a natural glycoside that is part of the flavone class. The compound is found in a multitude of vegetables and fruits such as wheat sprouts, onions, tea, and oranges [55]. Prior studies have exhibited that tumor proliferation, invasion, and tumor growth in prostate cells are inhibited by apigenin [56]. Moreover, apigenin has demonstrated an inhibitory effect on pancreatic cancer cell proliferation as well as the migration and invasion of A2780 human ovarian cancer cells [57]. Apigenin may serve as therapeutic agent in the prevention of OS cancers.

The anti-cancer properties of apigenin were demonstrated with U2OS cells, which underwent apoptotic induction as well as xenograft tumor growth inhibition. Apigenin demonstrated a marked effect on impairing proliferation of OS cells in a time- and dose-dependent manner in U2OS and MG-63 cells. Impairing cellular proliferation implied that apigenin was able to inhibit the survival of both cell lines. The cells were arrested in the G0/G1 phase after 24 h, indicating that the decrease in proliferation was in part attributed to cell cycle arrest. Apigenin was found to also inhibit the invasion of both U2OS and MG-63 cells. The inhibition appears to be a result of downregulating the expression β-catenin in the Wnt signaling pathway. The inhibition was evident by a reduction in β-catenin (Fig. 3b). Conversely, overexpression of β-catenin reversed the inhibiting effect of apigenin [57].

### Cyclopamine

Cyclopamine is a steroidal alkaloid that has shown ability to antagonize numerous cancers including breast cancers, prostate cancers, gastrointestinal cancers, and OS [31, 58–60]. The compound is derived from the corn lily (*Veratrum californicum*) [61]. When cyclopamine binds to the receptor smoothened (SMO), it prevents further signal transduction to the target gene GLI5 [31] (Fig. 4b). This inhibition has demonstrated interesting results in numerous experimentations dealing with tumors that are dependent on the Hh signaling pathway.

Warzecha et al. demonstrated that treating OS cells with cyclopamine resulted in a moderate reduction in the proliferation of the cells, depicted by viability assay. However, the anti-proliferative effect of cyclopamine was not due to the compound being a steroidal alkaloid but rather to the receptor effect on Hh signaling pathway. This notion was deduced by treating OS cells with another steroidal alkaloid known as tomatidine. This particular agent in contrast to cyclopamine lacked receptor activity against Hh signaling pathway. The tomatidine-treated cells had a proliferation of 51.6% compared to 18.5% from cyclopamine [31].

Researchers were then able to prove the cytotoxic effect of cyclopamine on OS cells in vivo. In the experiments, 5 × 10^5^ Os-50 cells were injected into the tail veins of young mice. Pulmonary metastasis in the controlled group of mice was significantly increased compared to the mice group who were treated with cyclopamine. Immunohistochemistry with Ki-67 antibody was comparatively higher in the cyclopamine group compared to the control group, implying a decreased percentage of immunoreactive metastatic cells being stained after cyclopamine treatment [31].

### Sulforaphane

Another natural compound that has been investigated for OS treatment is sulforaphane (SFN). This compound is a member of the isothiocyanate family and obtained from cruciferous vegetables including Brussels sprouts, cabbage, and broccoli [62]. Sawai et al. evaluated the effect of SFN and radiation treatment on LM8 murine OS cells. The cells were cultured with multiple concentrations of SFN that resulted in increased cell populations in G2/M phase. The combination of SFN and 2 Gy of radiation suppressed ERK and AKT phosphorylation. It was also discovered that SFN induced apoptosis through G2/M phase arrest and inhibited the activation of ERK and AKT [63] (Fig. 5). An additional study reported that SFN contributed to genomic instability in MG63 OS cell lines as evidenced by an increase in DNA breaks, nuclear and mitotic abnormalities, and clastogenicity. Loss of viability was evident by increased formation of micronuclei and apoptotic bodies [64]. SFN may prove to be a promising molecular targeting chemotherapeutic agent for OS cancers [63].

## Conclusions

Osteosarcoma continues to be a challenging cancer to treat, and there has been a notable lack of progress in survival statistics for this aggressive bone cancer. Progress has stalled in part due to the lack of knowledge of OS pathogenesis. Historically, the lack of understanding of cellular mediators involved in proliferation and invasion of OS impaired our ability to target those mediators. As a result, the same backbone of chemotherapy has continued to remain the primary treatment strategy. The overall 5-year event free survival of pediatric patients with metastatic OS has been poor at 30% [6]. Simultaneously, there has been an eruption of scientific research investigating signaling pathways that appear to play crucial roles in tumor survival and renewal capacity. Many of these signaling pathways appear to be susceptible to targeting with natural compounds. These natural compounds have the potential to target multiple aberrant pathways in OS. Numerous in vitro and in vivo studies have demonstrated that these phytochemicals can modulate the signal pathways of OS. These various phytochemicals have already demonstrated considerable efficacy in a variety of other cancer types. Given the extraordinary lack of progress seen in OS clinical trials that continue to use various combinations of cytotoxic chemotherapy, it is time we look closer at these targeted agents and natural compounds. We need to quickly elucidate their mechanisms of action and safety profiles to push them into larger clinical trials for upfront therapy, so that we can finally make substantial advancements in treating this aggressive cancer.

## Abbreviations

(EBP50): ERM-binding phosphoprotein 50
(NP-1): Nucleoprotein interactor 1
4E-BP1: 4E-binding protein 1
ADAM: A disintegrin and metalloprotease
ALDH: Aldehyde dehydrogenase
APH1: Anterior pharynx defective 1
BCl-2: B cell lymphoma 2
C-ERMAD: Carboxy-ERMAD
CK1α: Casein kinase 1 alpha 1
DATS: Diallyl trisulfide
Deptor: DEP domain-containing mTOR-interacting protein
DHh: Desert hedgehog
Dll-1: Delta-like-1
Dll-3: Delta-like-3
Dll-4: Delta-like-4
DNA-PK: DNA-dependent protein kinase
Dsh/DV1: Dsh/DV1
DSL: Delta/Serrate/Lag-2
eIF4E: Eukaryotic translation initiation factor 4E
EMT: Epithelial-to-mesenchymal transition
ERM: Ezrin/radixin/moesin
FOXO: Forkhead box O1
GDI: Guanosine diphosphate inhibitor
GDP: Guanosine diphosphate
GEF: GDP/GTP exchange factor
GLI1: GLI family zinc finger 1
GLI2: GLI family zinc finger 2
GLI3: GLI family zinc finger 3
GLI5: GLI family zinc finger 5
GP130: Glycoprotein 130
GSI: γ-Secretase inhibitors
GSK3β: Glycogen synthase 3β
GTP: Guanosine triphosphate
GβL: LST8/G-protein β-subunit like protein
Hes: Hairy and enhancer of split
Hey: Hes-related family BHLH transcription factor with YRPW motif
Hh: Hedgehog
IHh: Indian hedgehog
IL-6: Interleukin-6
ILK: Integrin-linked kinase
Jag-1: Jagged-1
Jag-2: Jagged-2
JAK: Janus tyrosine kinase
MAFK: v-maf avian musculoaponeurotic fibrosarcoma oncogene homolog K
MAML: Mastermind-like proteins
Mcl1: Myeloid cell leukemia 1
MDM2: Mouse double minute 2 homolog
miRNAs: MicroRNAs
MMP-2: Matrix metallopeptidase 2
MMP-9: Matrix metallopeptidase 9
mSIN1: Mammalian stress-activated protein kinase interacting protein
mTOR: Mammalian target of rapamycin
N-ERMAD: N-terminal ezrin/radixin/moesin (ERM) associated domain
NEXT: Notch extracellular truncation
NF-κB: Nuclear factor-κB
NICD: Notch intracellular domain
NKD2: Naked cuticle homolog-2 gene
NRR: Negative regulatory region
PDK1: Phosphoinositide-dependent kinase 1
PEN-2: Presenilin enhancer-2
PIP2: Phosphatidylinositol 4, 5-biphosphate
PIP3: Phosphatidylinositol 3,4,5-triphosphate
PRAS40: Proline rich AKT substrate
PTCH1: Patched 1
PTCH2: Patched 2
Raptor: Regulatory associated protein of mTOR
RBPJ/CBF1: Recombination signaling binding protein of hairless-J
Rheb: Ras homolog enriched in brain
RLN2: H2 relaxin
ROS: Reactive oxygen species
S6K: S6 kinases
Ser473: Serine 473
SFN: Sulforaphane
Shc: Src homology 2 domain-containing
SHh: Sonic hedgehog
sIL-6R: Soluble form of IL-6R
SMO: Smoothened
SOCS: Suppressors of cytokine signaling
STAT: Signal transducer and activator of the transcription
TACE/ADAM17/CD156q: Tumor necrosis factor-alpha converting enzyme
Thr308: Threonine 308
Tsc: Tuberous sclerosis complex
TYK2: Tyrosine kinase 2
VEGF: Vascular endothelial growth factor
β-Trcp: Beta-transducin repeat containing E3 ubiquitin protein ligase

## References

[1] Messerschmitt PJ, Garcia RM, Abdul-Karim FW, Greenfield EM, Getty PJ (2009). Osteosarcoma. J Am Acad Orthop Surg.

[2] Klein MJ, Siegal GP (2006). Osteosarcoma: anatomic and histologic variants. Am J Clin Pathol.

[3] Picci P (2007). Osteosarcoma (osteogenic sarcoma). Orphanet J Rare Dis.

[4] Botter SM, Neri D, Fuchs B (2014). Recent advances in osteosarcoma. Curr Opin Pharmacol.

[5] Jaffe N, Puri A, Gelderblom H (2013). Osteosarcoma: evolution of treatment paradigms. Sarcoma.

[6] Bacci G, Rocca M, Salone M, Balladelli A, Ferrari S, Palmerini E, Forni C, Briccoli A (2008). High grade osteosarcoma of the extremities with lung metastases at presentation: treatment with neoadjuvant chemotherapy and simultaneous resection of primary and metastatic lesions. J Surg Oncol.

[7] Zhang J, Yu XH, Yan YG, Wang C, Wang WJ (2015). PI3K/Akt signaling in osteosarcoma. Clin Chim Acta.

[8] Kopan R, Ilagan MX (2009). The canonical Notch signaling pathway: unfolding the activation mechanism. Cell.

[9] Garg V, Muth AN, Ransom JF, Schluterman MK, Barnes R, King IN, Grossfeld PD, Srivastava D (2005). Mutations in NOTCH1 cause aortic valve disease. Nature.

[10] Gridley T (2003). Notch signaling and inherited disease syndromes. Hum Mol Genet.

[11] Ehebauer M, Hayward P, Martinez-Arias A (2006). Notch signaling pathway. Sci STKE.

[12] Okajima T, Matsuda T (2006). Roles of O-fucosyltransferase 1 and O-linked fucose in notch receptor function. Methods Enzymol.

[13] Sato T, Diehl TS, Narayanan S, Funamoto S, Ihara Y, De Strooper B, Steiner H, Haass C, Wolfe MS (2007). Active gamma-secretase complexes contain only one of each component. J Biol Chem.

[14] Mumm JS, Kopan R (2000). Notch signaling: from the outside in. Dev Biol.

[15] Leong KG, Karsan A (2006). Recent insights into the role of Notch signaling in tumorigenesis. Blood.

[16] Anderson ME (2016). Update on survival in osteosarcoma. Orthop Clin North Am.

[17] Tsuru A, Setoguchi T, Matsunoshita Y, Nagao-Kitamoto H, Nagano S, Yokouchi M, Maeda S, Ishidou Y, Yamamoto T, Komiya S (2015). Hairy/enhancer-of-split related with YRPW motif protein 1 promotes osteosarcoma metastasis via matrix metallopeptidase 9 expression. Br J Cancer.

[18] Sethi N, Dai X, Winter CG, Kang Y (2011). Tumor-derived JAGGED1 promotes osteolytic bone metastasis of breast cancer by engaging notch signaling in bone cells. Cancer Cell.

[19] Mu X, Isaac C, Greco N, Huard J, Weiss K (2013). Notch signaling is associated with ALDH activity and an aggressive metastatic phenotype in murine osteosarcoma cells. Front Oncol.

[20] Won KY, Kim YW, Kim HS, Lee SK, Jung WW, Park YK (2013). MicroRNA-199b-5p is involved in the Notch signaling pathway in osteosarcoma. Hum Pathol.

[21] Hughes DP (2009). How the NOTCH pathway contributes to the ability of osteosarcoma cells to metastasize. Cancer Treat Res.

[22] Modder UI, Oursler MJ, Khosla S, Monroe DG (2011). Wnt10b activates the Wnt, notch, and NFkappaB pathways in U2OS osteosarcoma cells. J Cell Biochem.

[23] Engin F, Bertin T, Ma O, Jiang MM, Wang L, Sutton RE, Donehower LA, Lee B (2009). Notch signaling contributes to the pathogenesis of human osteosarcomas. Hum Mol Genet.

[24] Zou Y, Yang J, Jiang D (2015). Resveratrol inhibits canonical Wnt signaling in human MG-63 osteosarcoma cells. Mol Med Rep.

[25] Komiya Y, Habas R (2008). Wnt signal transduction pathways. Organogenesis.

[26] MacDonald BT, Tamai K, He X (2009). Wnt/beta-catenin signaling: components, mechanisms, and diseases. Dev Cell.

[27] Wang DZ, Gao JF, Jing SF, Wei H, Huang XY, Li CD. Antitumor effect of docetaxel in osteosarcoma by the inhibition of Wnt signal channel. Drug Res (Stuttg). 2014;65(11):597–601. 10.1055/s-0034-1395597 25514117

[28] Zhao S, Kurenbekova L, Gao Y, Roos A, Creighton CJ, Rao P, Hicks J, Man TK, Lau C, Brown AM, et al. NKD2, a negative regulator of Wnt signaling, suppresses tumor growth and metastasis in osteosarcoma. Oncogene. 2015;34(39):5069–79. 10.1038/onc.2014.429 PMC4802362 25579177

[29] Wang R, Zheng J, Zhang DS, Yang YH, Zhao ZF (2015). Wnt1-induced MAFK expression promotes osteosarcoma cell proliferation. Genet Mol Res.

[30] Kumar RM, Fuchs B (2015). Hedgehog signaling inhibitors as anti-cancer agents in osteosarcoma. Cancers (Basel).

[31] Warzecha J, Dinges D, Kaszap B, Henrich D, Marzi I, Seebach C (2012). Effect of the Hedgehog-inhibitor cyclopamine on mice with osteosarcoma pulmonary metastases. Int J Mol Med.

[32] Rivera-Valentin RK, Zhu L, Hughes DP (2015). Bone sarcomas in pediatrics: progress in our understanding of tumor biology and implications for therapy. Paediatr Drugs.

[33] Song R, Tian K, Wang W, Wang L (2015). P53 suppresses cell proliferation, metastasis, and angiogenesis of osteosarcoma through inhibition of the PI3K/AKT/mTOR pathway. Int J Surg.

[34] Lu X, Zhao J, Li T, Huang M, Liang J, Wei W (2015). 5,7-Dihydroxy-4′-methoxyisoflavone induces apoptosis by inhibiting the ERK and Akt pathways in human osteosarcoma cells. Connect Tissue Res.

[35] Ma J, Huang H, Han Z, Zhu C, Yue B (2015). RLN2 is a positive regulator of AKT-2-induced gene expression required for osteosarcoma cells invasion and chemoresistance. Biomed Res Int.

[36] Jokinen E, Koivunen JP (2015). MEK and PI3K inhibition in solid tumors: rationale and evidence to date. Ther Adv Med Oncol.

[37] Yu Y, Luk F, Yang JL, Walsh WR (2011). Ras/Raf/MEK/ERK pathway is associated with lung metastasis of osteosarcoma in an orthotopic mouse model. Anticancer Res.

[38] Choi SD (2012). Ezrin is an essential marker for metastasis of gynecologic cancer. J Korean Soc Menopause.

[39] Ren L, Khanna C (2014). Role of ezrin in osteosarcoma metastasis. Adv Exp Med Biol.

[40] Zhang J, Zuo J, Lei M, Wu S, Zang X, Zhang C (2014). Ezrin promotes invasion and migration of the MG63 osteosarcoma cell. Chin Med J (Engl).

[41] Zhao DH, Zhu J, Wang WB, Dong F, Zhang Q, Fan HW, Zhang JZ, Wang YM (2014). Correlations of ezrin expression with pathological characteristics and prognosis of osteosarcoma: a meta-analysis. ScientificWorldJournal.

[42] Celik H, Hong SH, Colon-Lopez DD, Han J, Saygideger Kont Y, Minas TZ, Swift M, Paige M, Glasgow E, Toretsky JA, et al. Identification of novel ezrin inhibitors targeting metastatic osteosarcoma by screening open access malaria box. Mol Cancer Ther. 2015;14(11);2497–507. 10.1158/1535-7163.MCT-15-0511 PMC4636458 26358752

[43] Tu B, Du L, Fan QM, Tang Z, Tang TT (2012). STAT3 activation by IL-6 from mesenchymal stem cells promotes the proliferation and metastasis of osteosarcoma. Cancer Lett.

[44] Cokic VP, Mitrovic-Ajtic O, Beleslin-Cokic BB, Markovic D, Buac M, Diklic M, Kraguljac-Kurtovic N, Damjanovic S, Milenkovic P, Gotic M, Raj PK. Proinflammatory Cytokine IL-6 and JAK-STAT Signaling Pathway in Myeloproliferative Neoplasms. Mediators Inflamm. 2015;2015:453020. 10.1155/2015/453020 PMC4602333 26491227

[45] McLornan D, Percy M, McMullin MF (2006). JAK2 V617F: a single mutation in the myeloproliferative group of disorders. Ulster Med J.

[46] Aggarwal BB, Sundaram C, Malani N, Ichikawa H (2007). Curcumin: the Indian solid gold. Adv Exp Med Biol.

[47] Chang R, Sun L, Webster TJ (2014). Short communication: selective cytotoxicity of curcumin on osteosarcoma cells compared to healthy osteoblasts. Int J Nanomedicine.

[48] Li Y, Zhang J, Ma D, Zhang L, Si M, Yin H, Li J (2012). Curcumin inhibits proliferation and invasion of osteosarcoma cells through inactivation of Notch-1 signaling. Febs J.

[49] Chang Z, Xing J, Yu X (2014). Curcumin induces osteosarcoma MG63 cells apoptosis via ROS/Cyto-C/Caspase-3 pathway. Tumour Biol.

[50] Li Y, Zhang J, Zhang L, Si M, Yin H, Li J (2013). Diallyl trisulfide inhibits proliferation, invasion and angiogenesis of osteosarcoma cells by switching on suppressor microRNAs and inactivating of Notch-1 signaling. Carcinogenesis.

[51] Ferrucci V, Boffa I, De Masi G, Zollo M. Natural compounds for pediatric cancer treatment. Naunyn Schmiedebergs Arch Pharmacol. 2016:389(2):131-49. 10.1007/s00210-015-1191-5 26650503

[52] Dandawate P, Padhye S, Ahmad A, Sarkar FH (2013). Novel strategies targeting cancer stem cells through phytochemicals and their analogs. Drug Deliv Transl Res.

[53] Varoni EM, Lo Faro AF, Sharifi-Rad J, Iriti M (2016). Anticancer molecular mechanisms of resveratrol. Front Nutr.

[54] Rusin M, Zajkowicz A, Butkiewicz D (2009). Resveratrol induces senescence-like growth inhibition of U-2 OS cells associated with the instability of telomeric DNA and upregulation of BRCA1. Mech Ageing Dev.

[55] Tutel’ian VA, Lashneva NV (2013). Biologically active substances of plant origin. Flavonols and flavones: prevalence, dietary sourses and consumption. Vopr Pitan.

[56] Shukla S, Kanwal R, Shankar E, Datt M, Chance MR, Fu P, MacLennan GT, Gupta S (2015). Apigenin blocks IKKalpha activation and suppresses prostate cancer progression. Oncotarget.

[57] Liu X, Li L, Lv L, Chen D, Shen L, Xie Z (2015). Apigenin inhibits the proliferation and invasion of osteosarcoma cells by suppressing the Wnt/beta-catenin signaling pathway. Oncol Rep.

[58] Zhu DM, Xue WL, Tao W, Li JC (2015). Effects of cyclopamine on the biological characteristics of human breast cancer MCF-7 cell line and its mechanism. Eur J Gynaecol Oncol.

[59] Lu ZY, Lu LD, Liang-Hong MA (2014). Effects of cyclopamine on the proliferation and apoptosis of LNCaP cells and expression of the PCA3 gene in human prostate cancer. Zhonghua Nan Ke Xue.

[60] Qualtrough D, Buda A, Gaffield W, Williams AC, Paraskeva C (2004). Hedgehog signalling in colorectal tumour cells: induction of apoptosis with cyclopamine treatment. Int J Cancer.

[61] Lee ST, Welch KD, Panter KE, Gardner DR, Garrossian M, Chang CW (2014). Cyclopamine: from cyclops lambs to cancer treatment. J Agric Food Chem.

[62] Fahey JW, Holtzclaw WD, Wehage SL, Wade KL, Stephenson KK, Talalay P (2015). Sulforaphane bioavailability from glucoraphanin-rich broccoli: control by active endogenous myrosinase. PLoS One.

[63] Sawai Y, Murata H, Horii M, Koto K, Matsui T, Horie N, Tsuji Y, Ashihara E, Maekawa T, Kubo T, Fushiki S (2013). Effectiveness of sulforaphane as a radiosensitizer for murine osteosarcoma cells. Oncol Rep.

[64] FerreiradeOliveira JM, Remedios C, Oliveira H, Pinto P, Pinho F, Pinho S, Costa M, Santos C (2014). Sulforaphane induces DNA damage and mitotic abnormalities in human osteosarcoma MG-63 cells: correlation with cell cycle arrest and apoptosis. Nutr Cancer.

[65] Margolis B, Skolnik EY (1994). Activation of Ras by receptor tyrosine kinases. J Am Soc Nephrol.

